# Anthropometric Influence on Preschool Children’s Physical Fitness and Motor Skills: A Systematic Review

**DOI:** 10.3390/jfmk9020095

**Published:** 2024-05-30

**Authors:** Markel Rico-González, Luca Paolo Ardigò, Ana P. Ramírez-Arroyo, Carlos D. Gómez-Carmona

**Affiliations:** 1 Department of Didactics of Musical, Plastic and Corporal Expression, University of the Basque Country, UPV-EHU, 48940 Leioa, Spain; 2 Department of Teacher Education, NLA University College, 0166 Oslo, Norway; 3 Optimization of Training and Sports Performance Research Group, Department of Didactics of Music, Plastic and Body Expression, Faculty of Sport Sciences, University of Extremadura, 10003 Caceres, Spain; aramirezjl@alumnos.unex.es (A.P.R.-A.); cdgomezcarmona@unex.es (C.D.G.-C.); 4 BioVetMed & SportSci Research Group, Department of Physical Education and Sport, Faculty of Sport Sciences, University of Murcia, 30720 San Javier, Spain

**Keywords:** health, early childhood, physiology, weight, obesity, physical fitness

## Abstract

Early childhood is a critical period for physical and motor development with implications for long-term health. This systematic review examined the relationship between anthropometric characteristics and measures of physical fitness and motor skills in preschool-aged children (typically 2–6 years). The search strategy was applied in four databases (PubMed, ProQuest Central, Scopus, and Web of Science) to find articles published before 11 April 2024. The results consistently demonstrated significant associations between anthropometric variables (height, weight, body mass index [BMI], body composition) and physical performance measures. Notably, height and mass were often better predictors of fitness status than BMI alone. Indicators of undernutrition (stunting, wasting) were negatively associated with motor development, emphasizing the importance of adequate nutrition. While some studies reported impaired fitness and motor skills among overweight/obese preschoolers compared to normal-weight peers, others found no differences based on weight status. Relationships between physical activity levels, anthropometrics, and motor outcomes were complex and inconsistent across studies. This review highlights key findings regarding the influence of anthropometric factors on physical capabilities in early childhood. Early identification of children with impaired growth or excessive adiposity may inform tailored interventions to promote optimal motor development and prevent issues like obesity. Creating supportive environments for healthy growth and age-appropriate physical activity opportunities is crucial during this critical developmental window.

## 1. Introduction

Early childhood, defined as the period from birth to 8 years old, is a critical stage for physical, cognitive, and socioemotional development [1]. During this time, children experience rapid growth and acquire fundamental skills that lay the foundation for future health and well-being [2]. One key aspect of early childhood development is the attainment of age-appropriate physical fitness and motor skills, which are essential for engaging in physical activity, exploring the environment, and developing social competencies [3].

Physical fitness refers to a set of attributes related to the ability to perform physical activities, including cardiorespiratory endurance, muscular strength and endurance, flexibility, and body composition [4]. Motor skills, on the other hand, encompass the coordinated movement patterns that enable the performance of everyday tasks, such as locomotor skills (e.g., running, jumping), object control skills (e.g., throwing, catching), and stability skills (e.g., balancing) [5]. Both physical fitness and motor skill proficiency have been linked to numerous health benefits in children, including improved cardiovascular function, bone health, cognitive development, and psychosocial well-being [6,7].

The development of physical fitness and motor skills in early childhood is influenced by a complex interplay of genetic, environmental, and behavioral factors [8]. Among these factors, anthropometric characteristics, such as height, weight, body mass index (BMI), and body composition, have been recognized as potential determinants of physical performance and motor development [9]. However, the nature and extent of these relationships remain unclear, with conflicting findings reported in the literature.

Several previous studies have explored related topics, such as the relationship between BMI and motor competence in preschoolers [10], the association between overweight/obesity and fundamental movement skills [11], or the influence of physical activity and sedentary behavior on physical fitness in this age group [12]. Rodrigues et al. [10] found a negative association between BMI and motor competence, suggesting that excess weight may hinder the development of motor skills. Similarly, Cattuzzo et al. [11] reported an inverse relationship between overweight/obesity and fundamental movement skills. Additionally, Poitras et al. [12] examined the relationship between physical activity, sedentary behavior, and physical fitness in preschoolers, emphasizing the importance of considering multiple anthropometric and behavioral factors in promoting healthy development. Finally, a longitudinal study conducted by Jaakkola et al. [13] found inconsistent evidence regarding the association between BMI and motor skills in children aged 3 to 12 years, highlighting the need for further research in this area.

While these studies provide valuable insights, they often focus on specific aspects or age groups. For instance, Rodrigues et al. [10] examined across childhood and adolescence or Cattuzzo et al. [11] focused on children aged 3–5 years. Age is an important factor that may influence the association between anthropometric characteristics and physical fitness and motor skills, as the rate and patterns of growth and development vary across different stages of early childhood [8]. In this sense, there is a need for a more comprehensive synthesis by examining the influence of various anthropometric characteristics, including height, weight, BMI, and body composition, on both physical fitness and motor skill development in the broader preschool population. By considering a wider range of anthropometric factors and their relationships with multiple domains of physical performance, this review seeks to elucidate the complex interplay between growth, body composition, and motor development during this critical yet dynamic period of rapid physical and neurodevelopmental changes [6,9].

Therefore, the present systematic review aims to address this gap by systematically summarizing the existing literature on the relationship between anthropometric characteristics (e.g., height, weight, BMI, body composition) and measures of physical fitness and motor skills in preschool-aged children (typically aged 2–6 years). The findings can inform tailored interventions and strategies to support optimal physical development, prevent obesity, and promote healthy lifestyles from an early age. Understanding these relationships is crucial for developing effective interventions and strategies to promote optimal physical development and prevent childhood obesity and the associated health risks.

## 2. Materials and Methods

### 2.1. Experimental Approach to the Problem

The PRISMA guidelines [14] and the guidelines for performing systematic reviews in sport sciences [15] were used for conducting this systematic review.

### 2.2. Information Sources

The search strategies were designed with the following characteristics:

Date: 11 April 2024.

Databases: PubMed, ProQuest Central, Scopus, and Web of Science.

### 2.3. Search Strategy

The P (Population), I (intervention), C (comparison), and O (Outcomes) strategy was applied for designing the search strategy, as suggested by those guidelines used for conducting this systematic review:

(*preschool* OR *kindergarten* OR “*early childhood*”) AND (*anthropometric* OR *morphology*) AND (“*physical fitness*” OR “motor skill*”)

This search string was adapted for use across the PubMed, ProQuest Central, Scopus, and Web of Science databases. Controlled vocabulary searching was supplemented with keyword searching to enhance retrieval. The searches were run to capture studies with no other restrictions on date of publication, language, or study design.

Citation searches were also performed for key included studies. When full-text articles could not be obtained through institutional subscriptions or open access, attempts were made to contact the corresponding authors directly.

### 2.4. Inclusion/Exclusion Criteria

The search was run out by two authors, who identified the most relevant information from each of the papers. Then, the information (title, authors, date, and database) was downloaded to an excel document, where duplicates were selected and removed. If any relevant study was found that was not through the search strategy, it was added as “added from external sources”. Each of the articles was analyzed for this eligibility following the inclusion–exclusion criteria detailed in the Table 1.

### 2.5. Data Extraction

All the studies included in the excel document were checked to determine if each passed or not all the inclusion criteria. The process was independently conducted by the two authors. Any disagreements (5% of the total) on the final inclusion–exclusion status were resolved through discussion in both the screening and excluding phases and a final decision was agreed upon. In this discussion, both authors analyzed the article at the same time, looking for the exclusion criteria following all the criteria detailed in the Table 1, in the same order. This process was registered in the excel document. 

### 2.6. Methodological Quality

To evaluate the risk-of-bias, each of the included articles was evaluated using an adapted version [16] from the original version developed by Law et al. [17]. This scale was composed by 16 items, detailed in Table 2.

## 3. Results

### 3.1. Identification and Selection of Studies

In total, 241 original articles were initially found, from which 60 were duplicated. The remaining 181 original articles were checked against the inclusion/exclusion criteria. After this analysis, 155 articles were eliminated because they did not meet one of the inclusion criteria detailed in the Table 1:

- Inclusion criteria number one: 77
- Inclusion criteria number two: 13
- Inclusion criteria number one: 65

**Figure 1:**
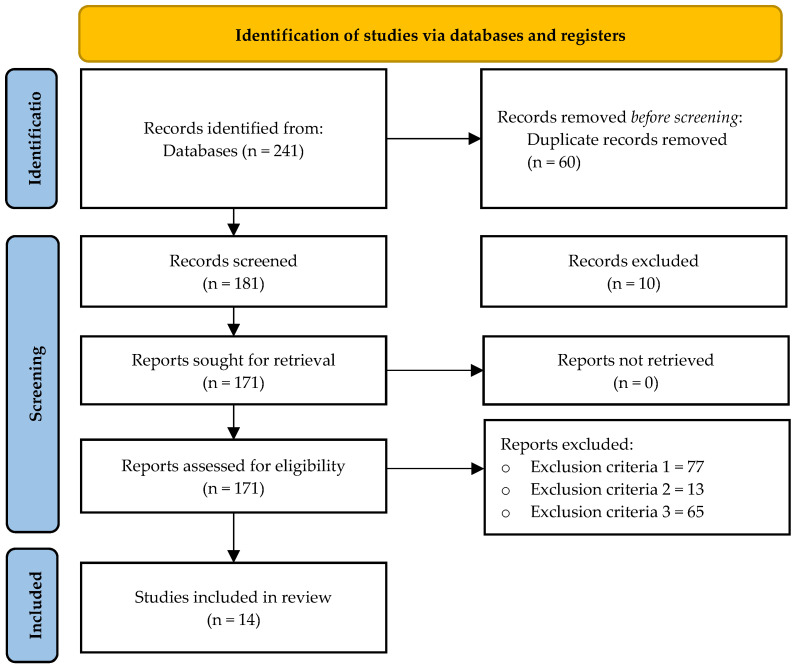
Flow diagram of the systematic review.

After this process, 14 articles meet all the inclusion criteria. All of them were evaluated for risk-of-bias, and then, included in Table 3, for extracting all the relevant information (Figure 1).

### 3.2. Quality Assessment

Table 2 presents a methodological assessment of multiple studies, evaluating their quality based on specific criteria. Most studies scored between 13 and 16 out of 16 points, indicating generally high methodological quality, although some variations in scores were observed, suggesting differences in methodological rigor among the studies.

### 3.3. Study Characteristics

Table 3 summarizes 14 studies that investigated the relationships between anthropometric characteristics, physical activity levels, motor skills, and physical fitness among preschool children. The key variables examined across these studies included height, weight, body mass index, skinfold thicknesses, circumferences, physical activity measures, and fitness test scores.

Several studies aimed to develop equations or critical values to predict physical fitness status from simple anthropometric measures like height and weight [18,19]. Others explored how anthropometric indicators of growth and nutritional status were associated with motor skill development [21,30] or risk of overweight/obesity [25,26,27].

The influence of weight status on performance in motor skills and fitness tests was examined [25,26,31], as was the relationship between physical activity, sedentary behavior, body composition, and fitness [23,24,29]. Some studies also described norms or percentiles for motor development scores relative to anthropometric measures [22,28].

## 4. Discussion

The results demonstrated significant associations between anthropometric variables such as height, weight, BMI, waist circumference, and measures of physical fitness and motor skills in preschool children. These findings are consistent with previous research that have reported inverse relationships between excess weight/obesity and motor competence [10,11] and physical fitness [12] in young children. However, the present review extends these findings by examining a broader range of anthropometric indicators and their relationships with multiple aspects of physical performance and motor development.

First, the results consistently demonstrate the importance of anthropometric measurements, particularly height, mass, and body composition, in influencing physical performance and motor development in preschool children. Several studies reported positive significant associations between these anthropometric variables and measures of physical fitness, such as muscular strength, endurance, and cardiorespiratory fitness [18,19,20,21,22]. In this sense, Kondrič et al. [18] found that height and mass were better predictors of fitness status than BMI or skinfold measurements, suggesting the potential value of these easily obtainable anthropometric indicators as screening tools in this age group. These findings are supported by studies suggesting BMI may not accurately capture musculoskeletal development importance for motor skills [32,33]. Additionally, studies found that stunting, underweight, and other indicators of malnutrition were negatively associated with motor development. [21,22,30]. These results align with previous evidence linking malnutrition to impaired growth and neurodevelopment [34,35]. This underscores the importance of adequate nutrition for supporting optimal physical and cognitive development and growth.

The influence of anthropometric characteristics on motor skills was also evident in the reviewed studies [21,22,23]. Sudfeld et al. [21] reported linear associations between height and motor development, while mass was linearly related to motor skills only in wasted children. Selvam et al. [22] derived age-specific norms for developmental milestones and found that stunted and underweight children exhibited significantly lower scores for communication and motor skills compared to their normal-weight counterparts. Similarly, Silventoinen et al. [19] observed associations between chest circumference and motor development, indicating the potential influence of prenatal and early postnatal factors on later motor abilities.

Another noteworthy finding from the reviewed studies was the relationship between anthropometric measures and weight status (e.g., overweight/obesity) in preschool children. Several studies reported impaired physical fitness and motor performance among overweight and obese preschoolers compared to their normal-weight counterparts [25,26,31]. For example, Agha-Alinejad et al. [25] reported that overweight and obese preschoolers demonstrated inferior performance in various fitness tests compared to their normal-weight peers. In contrast, De Toia et al. [26] found no significant differences in motor abilities between overweight/obese children and their normal/underweight counterparts, except for underweight children performing worse in flexibility tests aligning with inconsistent evidence reported in longitudinal studies [13]. The discrepancies between studies may be attributable to variations in study populations, measurement techniques, or the specific skills assessed [36,37]. These findings underscore the importance of early intervention and prevention strategies to promote healthy growth and development, as well as the potential implications for later health outcomes.

In addition, several studies examined the impact of specific conditions, such as motor delay or foot status, on anthropometric characteristics and motor performance. Hwang et al. [27] found that physical activity was a common predictor of higher BMI in children with and without motor delay. For instance, Kojić et al. [28] reported significant differences in motor status among preschoolers with varying foot statuses, suggesting that even subtle variations in physical characteristics can impact motor skill development. Collectively, these findings emphasize the importance of early identification and intervention for children with impaired growth, development, or specific conditions that may affect their physical fitness and motor skills.

Furthermore, the reviewed studies highlight the complex interplay between anthropometric characteristics, physical activity (PA), and fitness levels in preschool children. Serrano-Gallén et al. [29] reported associations between PA subcomponents (e.g., vigorous PA) and body composition and physical fitness, aligning with evidence linking higher activity levels to better fitness/motor skills [38,39]. On the other hand, some studies conducted by Bergqvist-Norén et al. [23] or Leppänen et al. [24] found no significant relationships between PA and weight status or motor skills. These discrepancies may be due to differences in activity measurement, age ranges, or other contextual factors [40,41]. Finally, it is important to note that the methodological quality of the included studies was generally high, with most studies scoring between 13 and 16 out of 16 points on the quality assessment scale. This indicates a relatively robust body of evidence, although some variations in methodological rigor were observed.

While the present systematic review provides valuable insights into the relationship between anthropometric characteristics and physical fitness and motor skills in preschool children, it is essential to acknowledge its limitations. First, the review focused specifically on preschool-aged children, limiting the generalizability of the findings to other age groups. Second, the search strategy and inclusion criteria may have missed potentially relevant studies, introducing potential publication bias. Third, the heterogeneity in study designs, outcome measures, and analytical approaches across the included studies hindered direct comparisons and meta-analytical synthesis. Finally, the review did not assess the risk of bias or study quality using a comprehensive, validated tool, potentially introducing bias in the interpretation of the findings.

## 5. Conclusions

The present systematic review provides compelling evidence on the influence of anthropometric characteristics, such as height, mass, and body composition, on physical fitness and motor skill development in preschool-aged children. The findings underscore the importance of early identification and intervention for children with impaired growth or development, as well as the potential implications for obesity prevention and promoting healthy lifestyles. This review highlights the complex interplay between anthropometric measures, physical activity, and fitness levels, suggesting the need for a holistic approach in supporting optimal growth and development during this critical period.

The findings from this systematic review have several practical applications for healthcare professionals, educators, and policymakers working with preschool-aged children. Early screening and assessment of anthropometric characteristics, physical fitness, and motor skills can aid in identifying children at risk for developmental delays or health issues. Tailored intervention programs that integrate nutritional rehabilitation, physical activity promotion, and motor skill development can be designed to address the specific needs of these children. Additionally, this review emphasizes the importance of creating supportive environments and implementing policies that encourage healthy growth, active lifestyles, and age-appropriate physical activity opportunities for preschoolers. Collaboration among stakeholders, including healthcare providers, educators, and families, is crucial to ensure a comprehensive approach to promoting optimal development and well-being in this critical stage of life.

## Figures and Tables

**Table 1:** Inclusion/exclusion criteria.

Topic	Inclusion	Exclusion	Search Coherence
**Population**	Children attending preschools (2–6 years) without special needs	Children who do not attend preschools, or children with special needs	Preschool OR kindergarten OR “early childhood”
**Intervention or exhibition**	Children involved in preschools	Children not involved in preschools	
**Results**	Outcomes that relates anthropometric characteristics (e.g., weight, height) and physical fitness or motor skills	Outcomes non related to anthropometric characteristics (e.g., weight, height), physical fitness, or motor skills	(anthropometric OR morphology) AND (“physical fitness” OR “motor skill*”)
**Study design**	-	-	-
**Other critics**	Original articles that have been peer-reviewed	Articles written without peers, reviewing the complete original text studies	-

**Table 2:** Methodological assessment of the included studies.

Reference	1	2	3	4	5	6	7	8	9	10	11	12	13	14	15	16	Score
Kondrič et al. [18]	1	1	1	1	0	1	1	1	1	1	1	1	0	1	1	0	13/16
Silventoinen et al. [19]	1	1	1	1	0	0	1	1	1	1	1	1	0	1	1	1	13/16
Morales-Gavilán et al. [20]	1	1	1	1	0	1	1	1	1	1	1	1	0	1	1	1	13/16
Sudfeld et al. [21]	1	1	1	1	0	0	1	1	1	1	1	1	0	1	1	1	13/16
Selvam et al. [22]	1	1	1	1	1	1	1	1	1	1	1	1	0	1	1	1	15/16
Bergqvist-Norén et al. [23]	1	1	1	1	0	1	1	1	1	1	1	1	1	1	1	1	15/16
Leppänen et al. [24]	1	1	1	1	1	1	1	1	1	1	1	1	1	1	1	1	16/16
Agha-Alinejad et al. [25]	1	1	1	1	1	1	0	0	1	1	1	1	0	1	1	0	14/16
De Toia et al. [26]	1	1	1	1	0	1	1	1	1	1	1	1	0	1	1	1	14/16
Hwang et al. [27]	1	1	1	1	0	1	1	1	1	1	1	1	0	1	1	0	14/16
Kojić et al. [28]	1	1	1	1	0	1	1	1	1	1	1	1	0	1	1	1	14/16
Serrano-Gallén et al. [29]	1	1	1	1	0	1	1	1	1	1	1	1	1	1	1	1	15/16
Worku et al. [30]	1	1	1	1	1	1	1	1	1	1	1	1	1	1	1	1	16/16
Cadenas-Sánchez et al. [31]	1	1	1	1	0	0	1	1	1	1	1	1	0	1	1	1	13/16

**Note:** Item 1: Was the study purpose stated clearly?; Item 2: Was relevant background literature reviewed?; Item 3: Was the design appropriate for the research question?; Item 4: Was the sample described in detail?; Item 5: Was sample size justified?; Item 6: Was informed consent obtained? (if not described, assume No); Item 7: Were the outcome measures reliable? (if not described, assume No); Item 8: Were the outcome measures valid? (if not described, assume No); Item 9: Was method described in detail?; Item 10: Were results reported in terms of statistical significance?; Item 11: Were the analysis methods appropriate?; Item 12: Was importance for the practice reported?; Item 13: Were any drop-outs reported?; Item 14: Were conclusions appropriate given the study methods?; Item 15: Are there any implications for practice given the results of the study?; Item 16: Were the limitations of the study acknowledged and described by the authors?

**Table 3:** Characteristics of the studies included in this systematic review.

Reference	Aim	Sample	Anthropometrical Variables	Influenced Variables		Result	Practical Applications
				Physical Fitness Variables	Motor Skills Variables		
Kondrič et al. [18]	To calculate and interpret linear and nonlinear relationships between simple anthropometric predictors and physical fitness criteria among preschoolers of both genders and to find critical values of the anthropometric predictors which should be recognized as the breakpoint of the negative influence on the physical fitness.	Nº: 413 Country: Croatia Years: 5.08	Height, mass, triceps, and subscapular skinfolds scores (anthropometry)	Sit and reach, sit-ups in one minute, standing long jump, and shuttle run test testing scores	n/a	Height and mass are far better predictors of the physical fitness status than body mass index (BMI) and sum of triceps and subscapular skinfolds.	In some cases, evident regression breakpoints were found (approximately 25 kg in boys), which should be interpreted as critical values of the anthropometric measures for the studied sample of subjects.
Silventoinen et al. [19]	To analyze how the development in length, mass, head circumference, and chest circumference over infancy is associated with motor development in early childhood, using a twin study design.	Nº: 740 Country: Japan Years: 0 ÷ 1	Height, mass, head circumference, and chest circumference scores (anthropometry)	n/a	Maintain head, roll over, sit alone, stand holding on, walk holding on, walk independently, and running testing scores	Smaller body size and rapid catch-up growth are associated with delayed motor development. When studying these associations within twin pairs and thus adjusting the results for gestational age as well as many other maternal and postnatal environmental factors, chest circumference showed the most robust association.	Chest circumference, rarely used in developed countries, can offer additional information on prenatal conditions relevant for further motor development not achieved by more traditional anthropometric measures.
Morales-Gavilán et al. [20]	To analyze the physical performance of preschool children based anthropometric variables.	Nº: 217 Country: Spain Years: 4 ÷ 5.9	Weight, height, triceps fold, arm circumference body mass index (BMI), brachial muscle area (MBA), and brachial fat area (AGB) scores (anthropometry)	n/a	Ball throw and horizontal jump testing scores	Significant positive correlations were verified between ball throwing and age, height, and AMB in both sexes (r = 0.13, 0.37), while in the horizontal jump, in addition to being related to age, height, and AMB, weight also showed a significant positive correlation (r = 0.11, 0.41). Children of both sexes with greater height and AMB showed better physical performance, and there were marked differences between both sexes (*p* < 0.05).	In addition to chronological age, the somatic variables of height and arm muscle area could serve as growth indicators to assess the physical performance of preschoolers of both sexes.
Sudfeld et al. [21]	To assess the association between anthropometric growth indicators across their distribution and determinants of malnutrition with development of Tanzanian children.	Nº: 1036 Country: Tanzania Years: 18 ÷ 36 months	Height and mass scores (anthropometry)	n/a	Fine and gross motor development scores (Bayley Scales of Infant Development III)	Height was linearly associated with motor development across the observed range in this population. Mass was linearly associated with motor development across the observed range only in wasted children.	Mild to severe chronic malnutrition was associated with increasing developmental deficits in Tanzanian children.
Selvam et al. [22]	To develop age-specific norms for developmental milestones using Vineland Adaptive Behavior Scales for apparently healthy children from 2 to 5 years from urban Bangalore, India, and to examine its association with anthropometric measures.	Nº: 412 Country: India Years: 2 ÷ 5.9	Height and mass scores (anthropometry)	n/a	Communication, daily living skills, socialization, and motor skills scores (Vineland Adaptive Behavior Scales)	Age-specific norms were derived. Stunted and underweight children had significantly lower developmental scores for communication and motor skills compared with normal children.	Early identification of children with impaired growth and development should be a critical component of childhood intervention programs.
Bergqvist-Norén et al. [23]	To examine patterns and changes of accelerometer-measured physical activity (PA) over time in two to six-year-old children in relation to parental PA, socioeconomic status, sex, weight status, and motor skills.	Nº: 106 Country: Sweden Years: 2 ÷ 6	Height and mass scores (anthropometry)	PA scores (accelerometry)	Motor skills testing scores	No significant relationships were found between child PA and weight status or motor skills.	Factors other than weight status and motor skills influence PA in children.
Leppänen et al. [24]	To examine associations of PA and sedentary behavior (SB) with body composition and physical fitness in healthy Swedish 4-year-old children.	Nº: 307 Country: Sweden Years: 4.48 ± 0.15	Body composition scores (air-displacement plethysmography)	PA, SB, and physical fitness scores (cardiorespiratory fitness, lower and upper body muscular strength, and motor fitness; PREFIT fitness test battery)	n/a	Time spent on vigorous-intensity PA (VPA) was associated with higher fat-free mass index and better physical fitness.	Promoting VPA may be important to improve childhood body composition and physical fitness already at an early age.
Agha-Alinejad et al. [25]	To compare the prevalence of overweight and obesity among preschoolers living in the capital of Iran and to determine relationships between overweight and obesity and selected motor- and health-related fitness parameters.	Nº: 381 Country: Iran Years: 5–6	Height, body mass, WC, WHR, WHtR, and PBF scores (anthropometry)	Sit-and-reach, modified sit-ups, modified pull-ups, the 4 m × 9 m shuttle run, the 20 m sprint test and the 20 m multistage shuttle run test testing scores	n/a	Significant correlations were found between modified pull-ups test and body mass, body mass index, WC, WHR, WHtR, and PBF in boys and modified pull-ups and modified sit-ups tests were significantly correlated with body mass, BMI, WC, WHR, WHtR, and PBF in girls. Compared with their counterparts, overweight and obese boys demonstrated inferior performance in modified pull-ups and predicted VO_2max_ and overweight and obese girls demonstrated inferior performance in modified pull-ups, modified sit-ups, 4 m × 9 m agility shuttle run and predicted VO_2max_.	The findings provided evidence to support the establishment of tailored physical fitness intervention programs to manage and prevent obesity in preschoolers.
De Toia et al. [26]	To examine the association between motor abilities and weight status in kindergarten children.	Nº: 1228 Country: Germany Years: 4.7 ± 1.0	Height and mass scores (anthropometry)	n/a	Speed strength, muscular endurance, coordination, flexibility, and speed scores (modified Karlsruher Motor Ability Screening Test)	Overweight and obese children did not differ from their normal and underweight counterparts except for underweight children which fared worse in flexibility.	The high number of overweight children and motor deficits suggests that preventive measures should start at this early age.
Hwang et al. [27]	To investigate the correlates of BMI and risk factors for overweight among 91 children with motor delay (MD) aged 9–73 months.	Nº: 91 Country: Taiwan Months: 40.9 ± 16.7	Height and mass scores (anthropometry)	PA scores (questionnaires)	n/a	BMI was correlated negatively with passive activity.	Children with MD and children without disabilities or chronic conditions share PA as a common potential predictor of higher BMI.
Kojić et al. [28]	To determine the differences in the manifestation of the motor status of normally fed preschool test subjects, classified into groups according to foot status.	Nº: 153 Country: Serbia Years: 3.9 ÷ 6.5	Mass, height, and foot status scores (anthropometry)	n/a	Running at 20 m from a high start, standing broad jump, backwards polygon, rectangular seated forward bend, plate tapping, sit-ups for 60 s, and bent arm hang testing scores	There was a statistically significant difference between groups of subjects with different foot statuses in the manifestation of motor status in all tests (namely, children with high arched feet showed better motor status than children with flat feet).	It is essential to stimulate the function and development of arches in preschool children.
Serrano-Gallén et al. [29]	To study the relationship between PA, fatness, and fitness in 3–6 years old boys and girls.	Nº: 150 Country: Spain Years: 3 ÷ 6	Mass, height, PBF, triceps skinfold, waist circumference, and waist-to-height ratio scores (anthropometry)	Fitness testing scores (handgrip strength, standing long jump, 4 × 10 m shuttle run and 20 m shuttle run; PREFIT fitness test battery), sit and reach test testing score and PA score (accelerometry)	n/a	PA subcomponents were not related to fatness. There were no significant differences in fitness tests between underweight, normal-weight, and over-weight children, except in handgrip strength.	The discrepancy about the relationship between PA and fatness between the results of the different research highlights the necessity for carrying out further studies that analyze the relationship between these 3 variables separately in each age group (3, 4, 5, and 6 years).
Worku et al. [30]	To ascertain the association of developmental outcomes and psychosocial factors after controlling nutritional indices.	Nº: 1638 Country: Ethiopia Months: 44.6 ± 21.2	Mass and height scores (anthropometry)	n/a	Development in fine and gross motor skills scores (Ages and Stages Questionnaires: Social-Emotional)	Stunting and underweightness were negatively associated with the development of fine and gross motor skills.	Intervention should integrate nutritional rehabilitation.
Cadenas-Sánchez et al. [31]	To describe anthropometric and physical fitness characteristics of low-income Chilean preschool children and to examine whether weight status influences children’s performance on fitness tests.	Nº: 434 Country: Chile Years: 5.48 ± 0.31	Mass, height, and WC scores (anthropometry)	Handgrip strength test, standing long jump, and 20 m sprint testing scores	n/a	Compared with normal-weight children, overweight/obese boys and girls were heavier and had greater waist circumference, were taller and showed higher performance in handgrip strength but not in standing long jump nor 20 m sprint.	Screening physical fitness levels in overweight/obese preschool children could be an important tool in order to design an efficacy PA programme.

**Note:** BMI = body mass index; n/a = no detailed; PA = physical activity; PBF = percentage of body fat; WC = waist circumference; WHtR = waist-to-height ratio.
